# Synthesis of silica nanoparticles from Vietnamese rice husk by sol–gel method

**DOI:** 10.1186/1556-276X-8-58

**Published:** 2013-02-06

**Authors:** Van Hai Le, Chi Nhan Ha Thuc, Huy Ha Thuc

**Affiliations:** 1 Laboratory of Polymer, Faculty of Chemistry, University of Science-National University of HoChiMinh City (VNU-HCM), 227 Nguyen Van Cu, Ward 4, District 5, HoChiMinh City, 70250, Vietnam; 2 Laboratory of Polymer and Composite Materials, Faculty of Materials Science, University of Science-National University of HoChiMinh City (VNU-HCM), 227 Nguyen Van Cu, Ward 4, District 5, HoChiMinh City, 70250, Vietnam

**Keywords:** Rice husk ash, Silica nanoparticles, Sol–gel method, CTAB

## Abstract

Silica powder at nanoscale was obtained by heat treatment of Vietnamese rice husk following the sol–gel method. The rice husk ash (RHA) is synthesized using rice husk which was thermally treated at optimal condition at 600°C for 4 h. The silica from RHA was extracted using sodium hydroxide solution to produce a sodium silicate solution and then precipitated by adding H_2_SO_4_ at pH = 4 in the mixture of water/butanol with cationic presence. In order to identify the optimal condition for producing the homogenous silica nanoparticles, the effects of surfactant surface coverage, aging temperature, and aging time were investigated. By analysis of X-ray diffraction, scanning electron microscopy, and transmission electron microscopy, the silica product obtained was amorphous and the uniformity of the nanosized sample was observed at an average size of 3 nm, and the BET result showed that the highest specific surface of the sample was about 340 m^2^/g. The results obtained in the mentioned method prove that the rice husk from agricultural wastes can be used for the production of silica nanoparticles.

## Background

Globally, approximately 600 million tons of rice paddies is produced each year. On an average, 20% of the rice paddy is husk, giving an annual total production of 120 million tons [1]. In Vietnam, the average output of the country is 42 billion tons per year, and this country is the second largest manufacturer of rice in the world. Rice husk (RH) is an agricultural waste material that should be eliminated. The chemical composition of RH is similar to that of many common organic fibers, containing cellulose, lignin, hemicelluloses, and silica, which is the primary component of ash. After burning, the organic composition is decomposed and rice husk ash (RHA) is obtained [1-3]. RHA is one of the most silica-rich raw materials containing about 90% to 98% silica and some amount of metallic impurities (after complete combustion) among the family of other agro-wastes [4-8]. It is important that the silica in RHA exists in the amorphous state and has high surface area [9-13]. Because of these features, silica has many applications, such as sources for synthetic adsorption materials [14-16], carriers, medical additives, fillers in composite materials, etc. [17,18], and demonstrates advantages when achieved at nanometer size.

Silica is a polymer of silicic acid consisting of inter-linked SiO_4_ units in a tetrahedral fashion with the general formula SiO_2_. In nature, it exists as sand, glass, quartz, etc. Naturally occurring silica is crystalline, whereas synthetically obtained silica is amorphous in nature. Silica used in chemical applications is synthesized from either silicate solution or silane reagents [19].

There are various methods to prepare silica nanoparticles. Adam et al. [20] synthesized spherical nanosilica from agricultural biomass as RH via the sol–gel method. The resulting silica particles were shown to be agglomerates with an average dimension of 15 to 91 nm. Jal et al. [21] synthesized nanosilica via the precipitation method, and the resulting nanosilica were found to have a particle size of 50 nm in dimension. However, the sol–gel technique [19,21-23] is the most common method for silica synthesis. It involves simultaneous hydrolysis and condensation reaction. In this process, a sol of sodium silicate or silicon alkoxide or halide gets converted into a polymeric network of gel. During silica synthesis by sol–gel process under certain conditions like restriction of gel growth, silica gets precipitated. In such preparation, the steps involved are coagulation and precipitation from silica solution. In the present investigation, we have focused our effort on preparing stable nanosilica from sodium silicate which was synthesized from Vietnamese rice husk using the sol–gel technique.

## Main text

### Materials

Rice husk from the natural rice source of Mekong Delta, Vietnam, was used. Sodium hydroxide, cetyltrimethylammonium bromide (CTAB), cetyl amine (CA), polyethylene glycol (PEG, 10,000), Arkopal, cethyl ammonium chloride (CAC), Aliquat 336, alkyl dimethyl benzyl ammonium chloride (ADBAC), cetylpyridiniumbromide (CPB), and cetyltrimethylammonium chloride (CTAC) were purchased from Merck (Darmstadt, Germany) and used as surfactant agents. Chlorhydric acid, sulfuric acid, and *n*-butanol were all purchased from Xilong (Guangzhou, China).

### Experimental procedure

#### Pretreatment of the RHA

The pretreatment of the RHA consisted of acid and thermal treatments. After treating the RH with 10% HCl and 30 wt.% sulfuric acid solution, the material was burned in a muffle furnace at 600°C for 4 h to remove all incorporated hydrocarbons.

An acid washing step was used to remove the small quantities of minerals prior to silica extraction from RHA in the following manner. The calcinated RHA (10 g) was acid-leached with 10% HCl and afterwards 30 wt.% sulfuric acid solution at 100°C for 2 h in a Pyrex three-neck round-bottom flask equipped with a reflux condenser in a hemispherical heating mantle. Then, the slurry was filtered and washed with distilled water for several times until the pH value equaled 7.

#### Preparation of sodium silicate solution

Sodium hydroxide solution (3.5 mol/L) was added to the pretreated RHA and boiled for 5 h in a Pyrex three-neck round-bottom flask equipped with a reflux condenser in a hemispherical heating mantle to dissolve the silica and to produce a sodium silicate solution. The solution was filtered and washed with boiling distilled water. The final solid sample was cooled to room temperature.

#### Synthesis of silica nanoparticles

Surfactant (2.0 wt.%) was dissolved in the water/butanol (1:1) solvent. Subsequently, RHA-derived sodium silicate was slowly added into the CTAB/water/butanol solution, and the mixture was stirred at 60°C. Then, 0.5 mol/L sulfuric acid solution was added gradually into the suspension in order to initiate the hydrolysis-condensation reaction at pH ~ 4. The resulting gel mixture was aged at 60°C for 8 h.

Then, 0.5, 1.0, 1.5, 2.0, 2.5, and 3.0 wt.% of CTAB were dissolved in the water/butanol solvent with 1:1 ratio. Subsequently, RHA-derived sodium silicate was slowly added to the CTAB water/butanol solution that was being stirred at 60°C. Then, 0.5 mol/L sulfuric acid solution was added gradually into the solution in order to initiate the hydrolysis-condensation reaction. The suspension was adjusted until the pH is 4. The resulting gel mixture was aged at different temperatures in the function of time. The aged silica gel was dispersed in butanol and washed with distilled water for several times. Nanosilica was calcinated at 550°C for 4 h in atmospheric condition to remove the surfactant. The final product was obtained and stored in desiccators before further characterizations.

## Discussion

The chemical compositions of the RHA before and after the treatment by acid were determined by adsorption atomic spectroscopy (AAS), and the results are presented in Table 1. Unlike conventional organic silicon compounds, the RHA is an agriculture waste, which contains several main extraneous components. The thermal and acid treatments are efficient, resulting in a material with high reduction in K_2_O, Al_2_O_3_, Fe_2_O_3_, CaO, and MgO contents. The silica (SiO_2_) in the RHA is not dissolved in the H_2_SO_4_ treatment. The silica nanoparticles are obtained via the following reactions:

NaOH + SiO_2_ → Na_2_SiO_3_ + H_2_O

Na_2_SiO_3_ + H_2_SO_4_ → SiO_2_ + Na_2_SO_4_ + H_2_O

**Table 1:** Chemical compositions of the RHA analyzed by AAS

**Component (wt.%)**	**K**_**2**_**O**	**Al**_**2**_**O**_**3**_	**Fe**_**2**_**O**_**3**_	**CaO**	**MgO**	**Na**_**2**_**O**	**SiO**_**2**_
Before treatment	0.39	0.48	0.15	0.73	0.55	0.12	96.15
After treatment	0.01	0.06	0.04	0.04	0.06	0.01	99.08

### Effect of surfactant on the particle size distribution of silica nanoparticles

**Figure 1:**
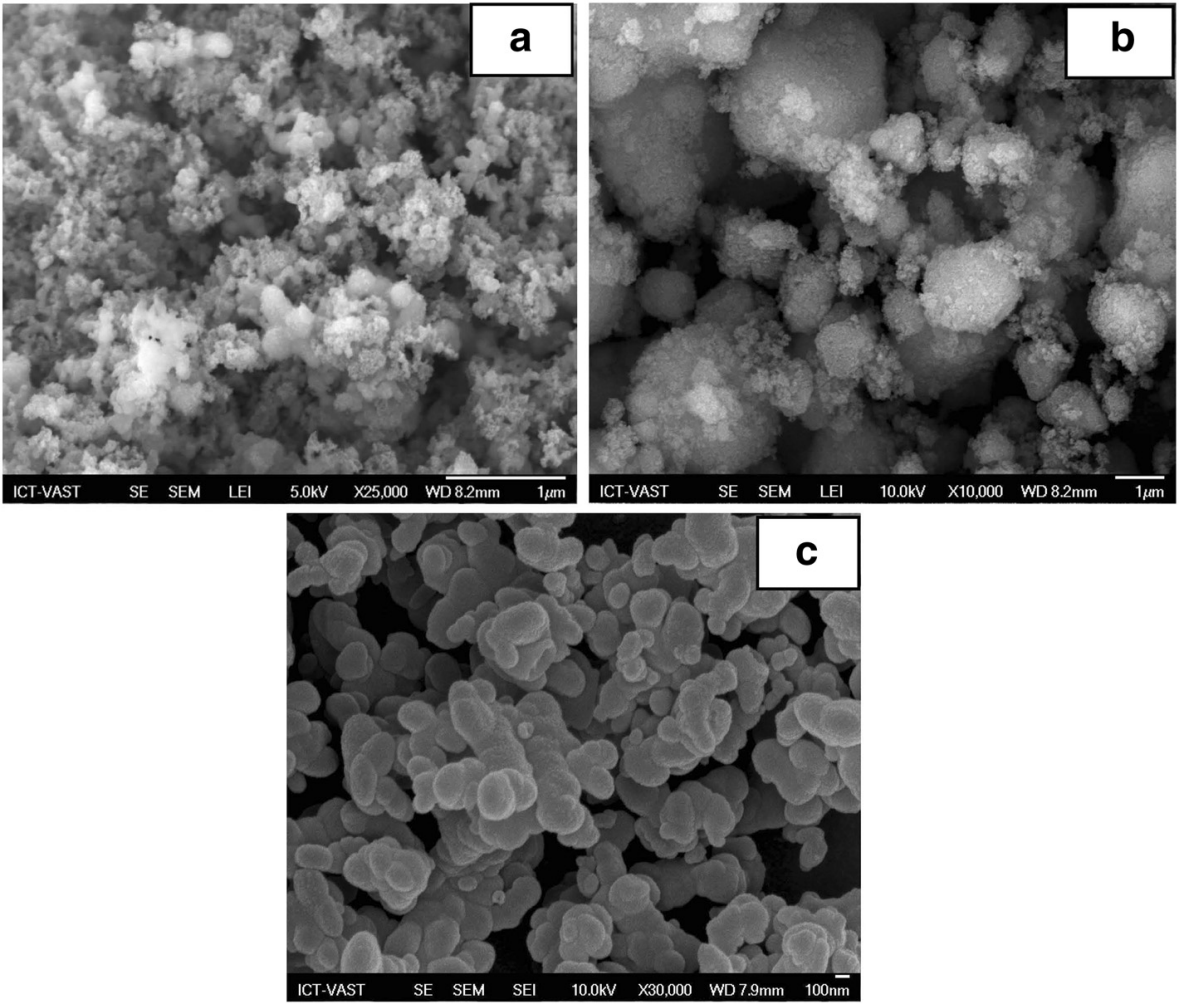
**SEM micrographs of silica nanoparticles obtained from surface-active substances.** CA (**a**), Arkopal (**b**), and PEG (**c**).

**Figure 2:**
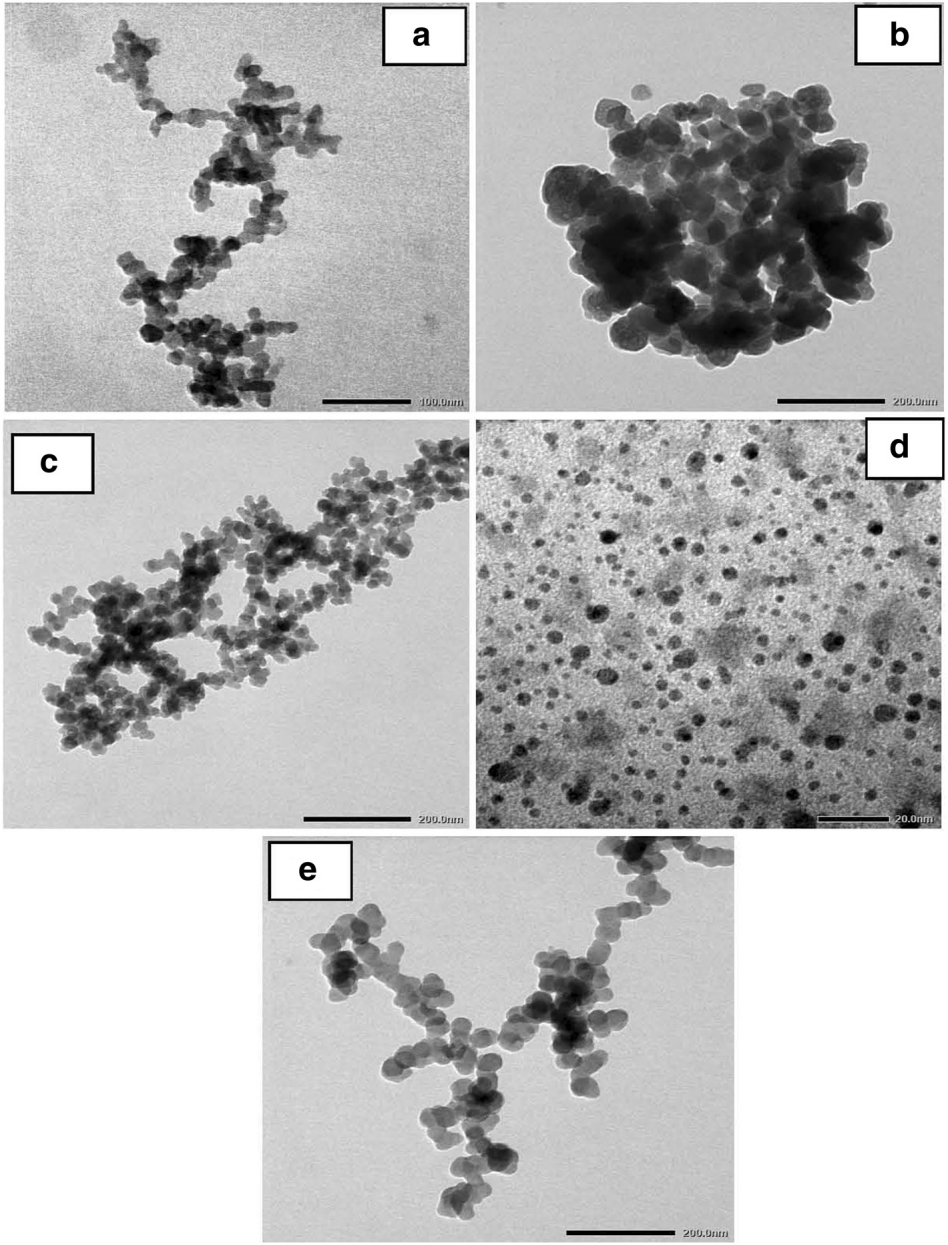
**TEM micrographs of silica nanoparticles obtained from surface-active substances.** CAC (**a**), ABDAC (**b**), Aliquat 336 (**c**), CTAB (**d**), and CPB (**e**).

In order to determine the influence of surface-active substances to the particle size, two groups of surface-active substances are investigated: The first group includes surface-active substances which are neutrally charged such as CA, PEG, and Arkopal. Scanning electron microscopy (SEM) images obtained are shown in Figure 1a,b,c. The second group includes cationic surface-active substances such as CAC, Aliquat 336, ADBAC, CPB, and CTAB. Transmission electron microscopy (TEM) images obtained are shown in Figure 2a,b,c,d,e. The concentration used for these surfactants is 2 wt.% with aging temperature at 60°C for 8 h.

The results show that the cationic surface-active substances do not coat uniformly the particle surface. In addition, due to the high surface energy and free OH groups on the silica surface which produce the hydrogen bond with water molecules, when the dispersed silica was isolated from the solvent, this hydrogen bond was also removed forming a Si-O-Si liaison and resulting to larger size particles which were agglomerated.

For surface-active substances of group 1, the mixture, after being synthesized, was dispersed completely in butanol phase and became transparent. The results show that the size distribution of silica particles is more uniform. In the case using Aliquat 336, ADBAC, and CPC, silica particles can be obtained in the form of a rope having an average particle size of 20 nm. Especially, when using the CTAB agent, the dispersion of the sample was much better with the smallest size of particles of about 2 to 4 nm. The result indicates that the CTAB surfactant has coated uniformly the surface of the material giving it much better dispersion in suspension.

### Effect of surfactant concentration on the particle size distribution of silica nanoparticles

In order to optimize the formation condition of silica nanoparticles, the effect of the CTAB concentration was investigated. The experiments were performed varying its concentration from 0 to 3 wt.% of total mass of silica, and the aging time and aging temperature condition are fixed at 8 h and 60°C, respectively.

**Figure 3:**
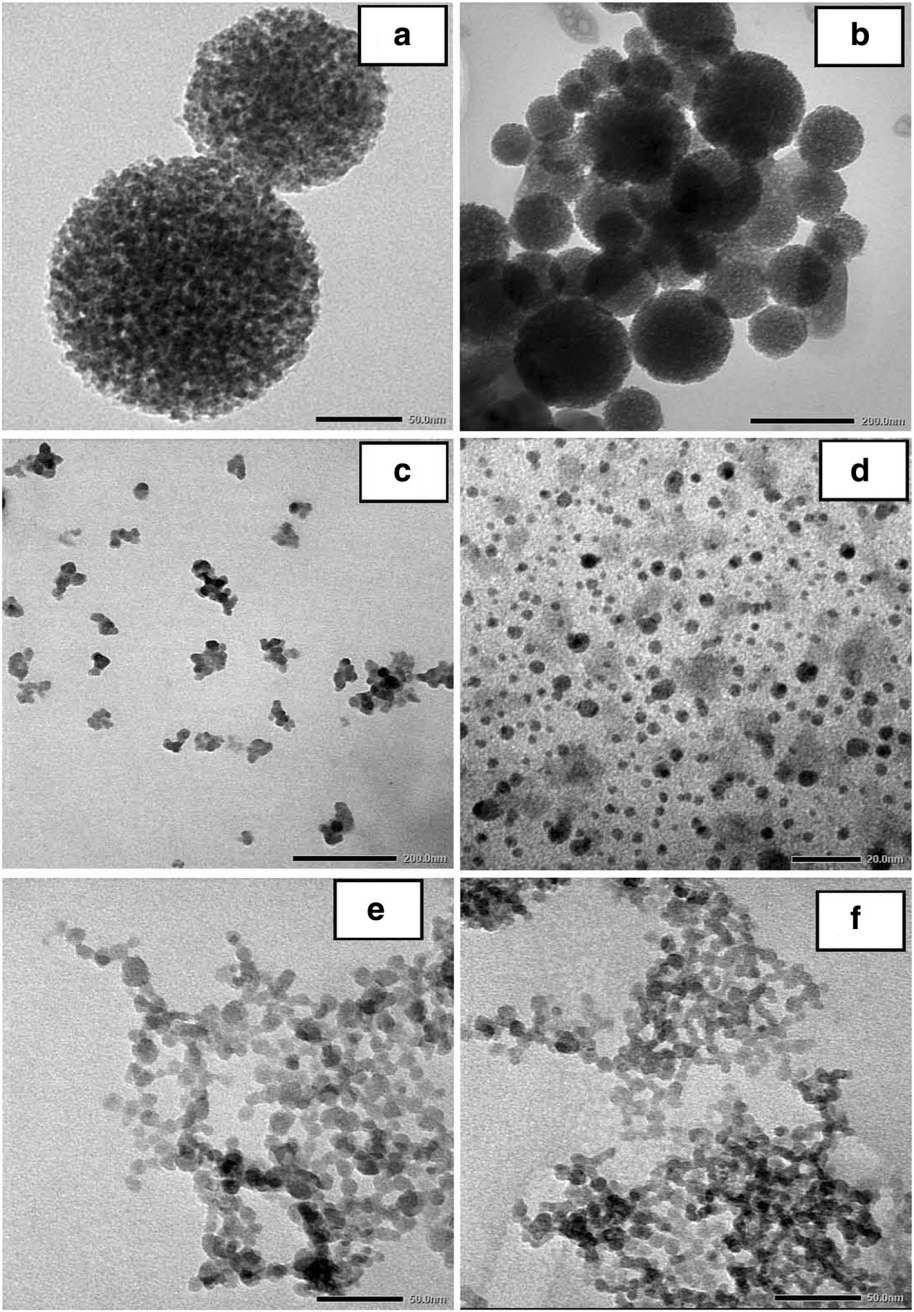
**TEM micrographs of silica nanoparticles obtained from CTAB.** 0.5 (**a**), 1.0 (**b**), 1.5 (**c**), 2.0 (**d**), 2.5 (**e**), and 3.0 wt.% (**f**).

**Figure 4:**
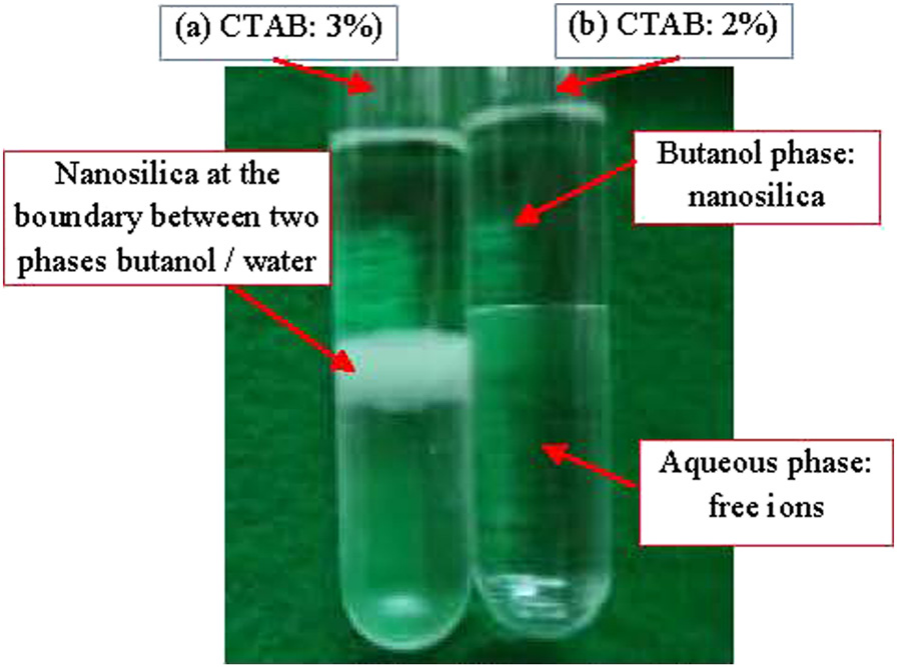
Silica nanoparticles dispersed in water/butanol.

The TEM micrographs of silica nanoparticles obtained at different CTAB concentrations are exhibited in Figure 3a,b,c,d,e,f. It can be clearly seen that the formed silica particles were seriously aggregated and the size ranged from a few nanometers to several hundred nanometers. In increasing the concentration of surfactant from 0.5 to 2.0 wt.% (Figure 3a,b,c,d), the particle size and uniform dispersion can be achieved. Above this concentration value of surfactant, the particle size becomes larger and causes aggregation. This suggests that 2 wt.% CTAB is the best surface-active substance to protect the surface of silica, in which silica nanoparticles are uniform (Figure 3d), which leads to the combination of silica and CTAB dispersed completely in the butanol solvent, as shown in Figure 4b (no polar hydrophilic agent). When the CTAB concentration was increased from 2.5 to 3.0 wt.% as shown in Figure 3e,f, the results show the appearance of small particles, while being distributed synchronously unclear, which tend to agglomerate, and silica nanoparticles were not distributed in the butanol solvent when the concentrations of CTAB were increased (Figure 4a).

### Effect of aging temperature and time on the particle size and its distribution of silica nanoparticles

Achieving the particle size and its distribution of silica nanoparticles depends on the stability of silica sol. Derjaguin [24] had distinguished three types of stability of colloidal systems: (1) phase stability, analogous to the phase stability of ordinary solutions; (2) stability of disperse composition, the stability with respect to the change in dispersity (particle size distribution); and (3) aggregative stability, the most characteristic for colloidal systems. Colloidal stability means that the particles do not aggregate at a significant rate. As explained earlier, an aggregate is used to describe the structure formed by the cohesion of colloidal particles. So, in this investigation, we have proved the effect of aging temperature and time on the stability of silica nanoparticles.

#### Effect of aging temperature

**Figure 5:**
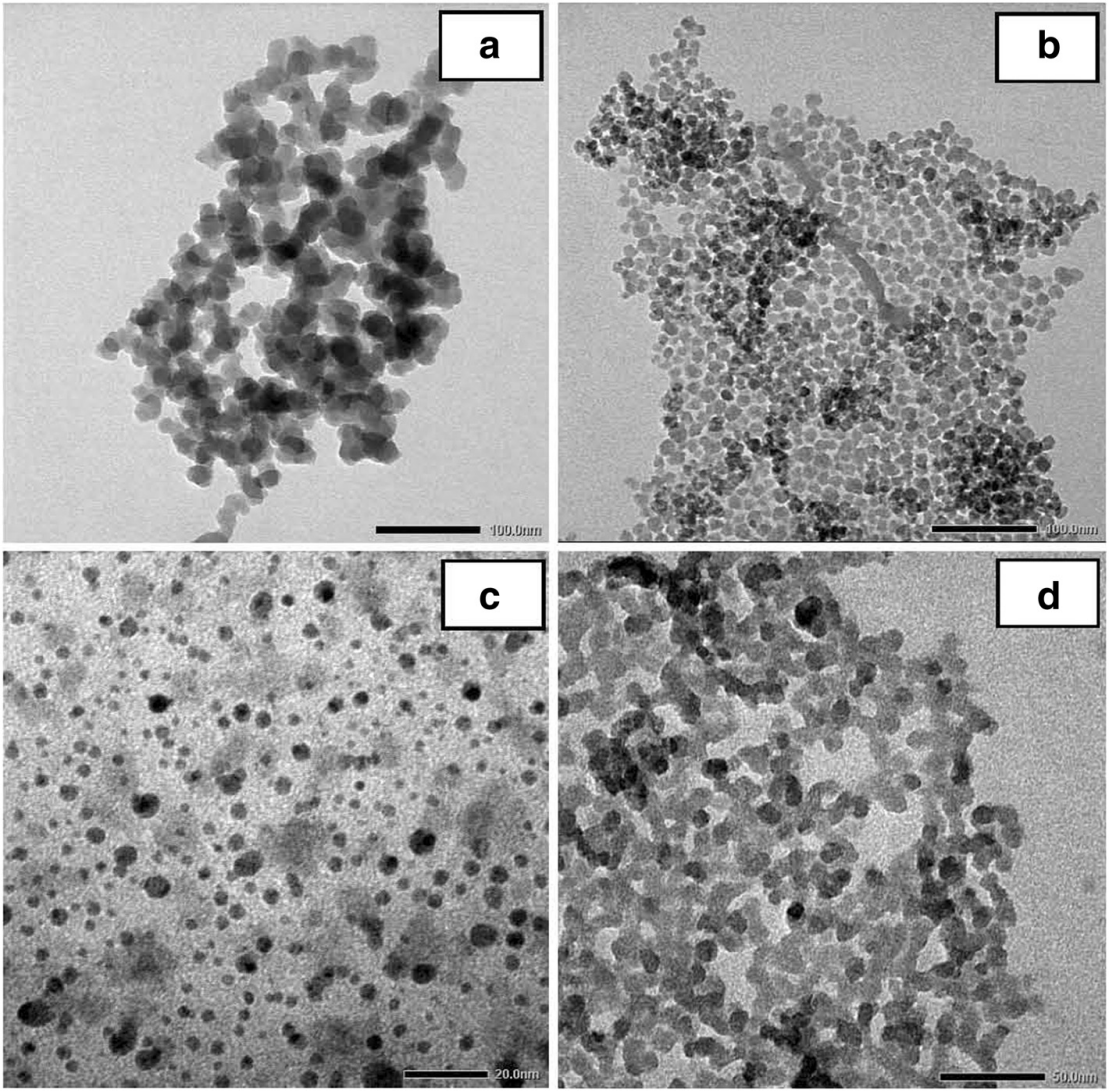
**TEM micrographs of silica nanoparticles obtained at different aging temperatures.** 30°C (**a**), 45°C (**b**), 60°C (**c**), and 80°C (**d**).

To optimize the formation condition of silica nanoparticles, the effect of aging temperature was investigated. The experiments were performed at different aging temperatures: 30°C, 45°C, 60°C, and 80°C, and the concentration of CTAB and aging time are fixed at 2.0 wt.% and 8 h, respectively. The TEM micrographs of silica nanoparticles obtained at different aging temperatures are exhibited in Figure 5a,b,c,d. The results obtained show that when the aging temperature changes, the dispersion states and sizes of silica nanoparticles also change and the best results of silica nanoparticles are achieved in the survey area at 60°C (Figure 5c). This suggests that the increase in temperature from 30°C to 60°C leads to increased interaction between the hydroxyl groups on the silica surface with CTAB. The result shows that the particle size has a better uniform distribution. However, when the aging temperature increased to 80°C, the CTAB molecules adsorbed on the surface of silica tend to desorption, which reduces the interaction between the molecules of the surface-active substance CTAB with hydroxyl groups on the silica surface, leading to reduced distribution of states of the silica nanoparticles and agglomeration between the particles via a bridge Si-O-Si.

Survey results on the influence of temperature on the particle size showed that the best condition in the survey area to obtain good dispersion and uniform particle size is at a temperature of 60°C with 2 wt.% CTAB.

#### Effect of aging time

**Figure 6:**
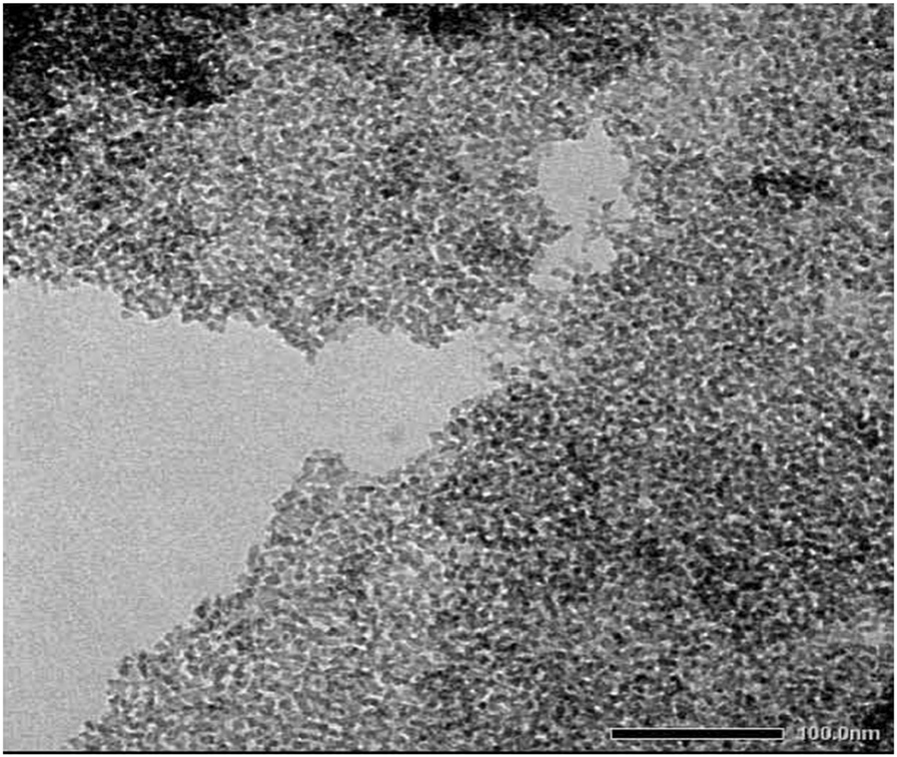
TEM micrograph of silica nanoparticles obtained at aging time 0 h.

**Figure 7:**
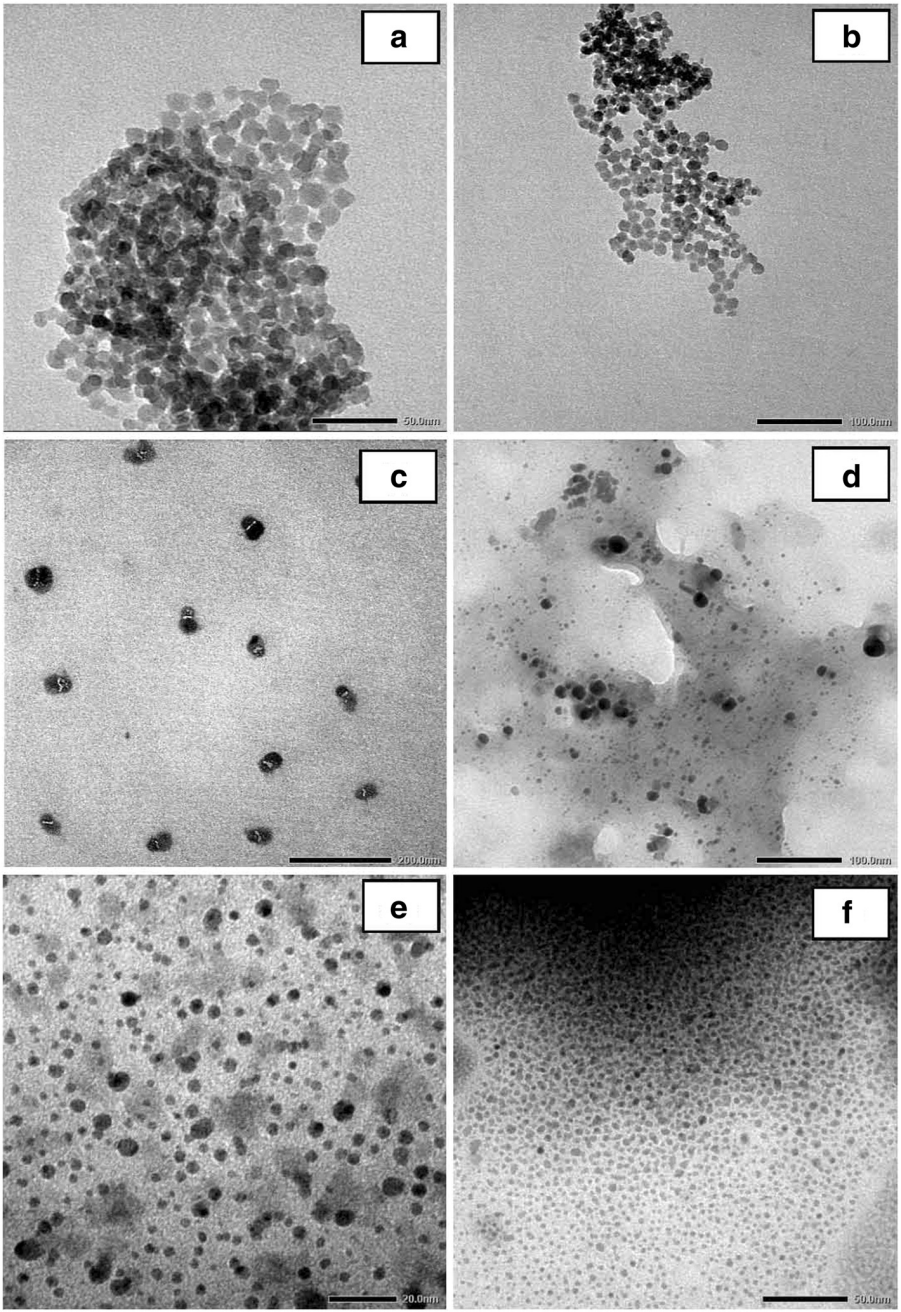
**TEM micrographs of silica nanoparticles obtained at different aging times.** 3 (**a**), 5 (**b**), 6 (**c**), 7 (**d**), 8 (**e**), and 12 h (**f**).

The aging time is then changed to check the role of different aging times in the particle size distribution. The experiments were performed varying the aging time at 0, 3, 5, 6, 7, 8, and 12 h, and the concentration of CTAB and aging temperature are fixed at 2.0 wt.% and 60°C, respectively. Figures 6 and 7a,b,c,d,e,f exhibit the TEM micrographs of silica nanoparticles formed in 2 wt.% CTAB surfactant with different aging times of 0, 3, 5, 6, 7, 8, and 12 h, respectively. From the TEM images, it is clearly seen that the particle size distribution becomes narrow with increasing aging time. When the aging time reached 8 h, the silica nanoparticles were uniformly dispersed in the solvents. It can be attributed to the fact that the aging time plays an important role in the particle size distribution. Aging is a process of dissolution and reprecipitation driven by differences in solubility. Based on the aging theory, during the aging process of silica gel, the smaller silica particles are dissolved and the silica particles are reprecipitated onto larger particles with the increase of aging time. As the aging time increased to 8 h, the silica gel reached dissolution equilibrium. So, the silica particles were uniformly dispersed in the solvents.

**Figure 8:**
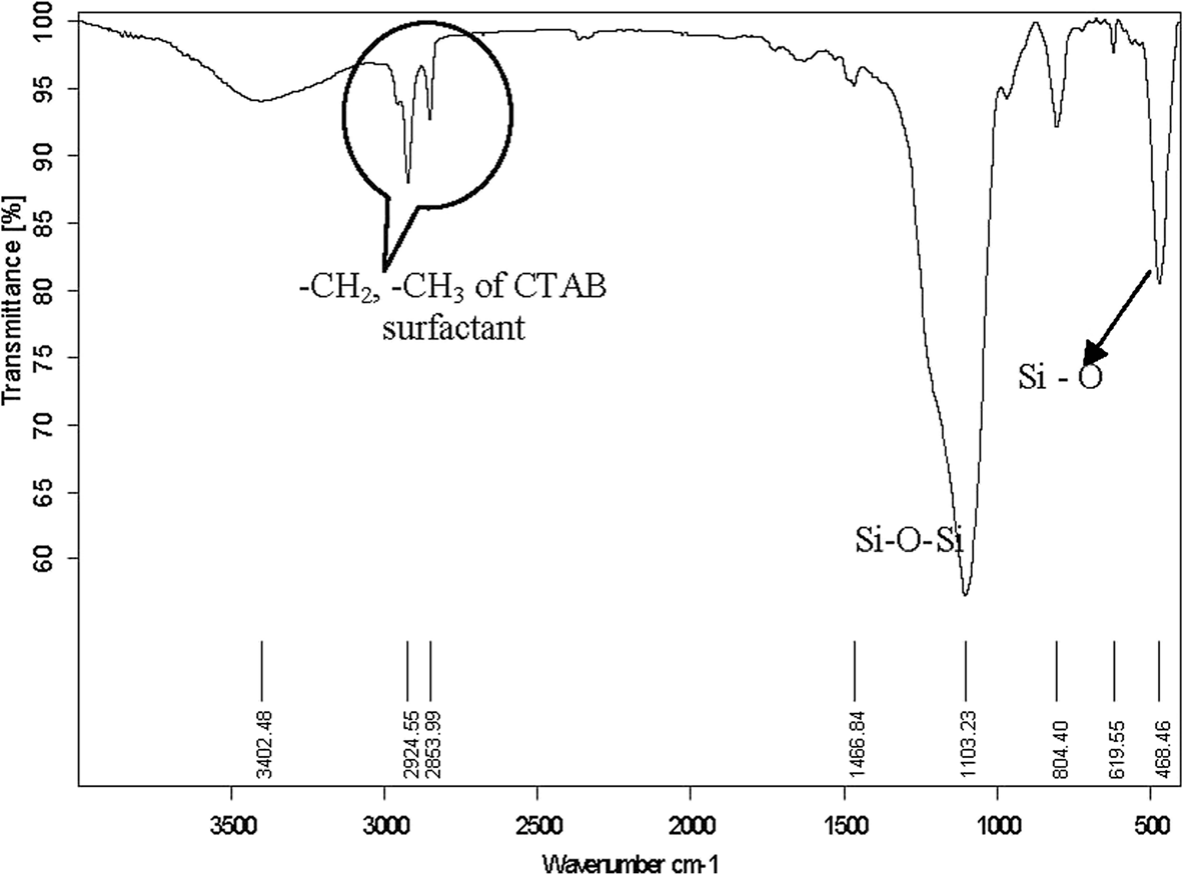
FT-IR spectra of the nanoparticles.

The Fourier transform infrared (FT-IR) spectra of the silica nanoparticles dried at 100°C are shown in Figure 8. The peaks at 1,103, 804, and 488 cm^−1^ are due to the asymmetric, symmetric, and bending modes of SiO_2_, respectively. The broad absorption band at 3,402 cm^−1^ and the peak at 1,466 cm^−1^ for the sample are due to the -OH groups. The absorption bands observed at 2,924 and 2,853 cm^−1^ are due to the bending of -CH_2_ and -CH_3_ of the CTAB surfactant. The FT-IR spectra show C-H peaks at 2,924 and 2,853 cm^−1^, clearly indicating the organic modification of the nanoparticle surface and the silica nanoparticle obtained in amorphous state.

**Figure 9:**
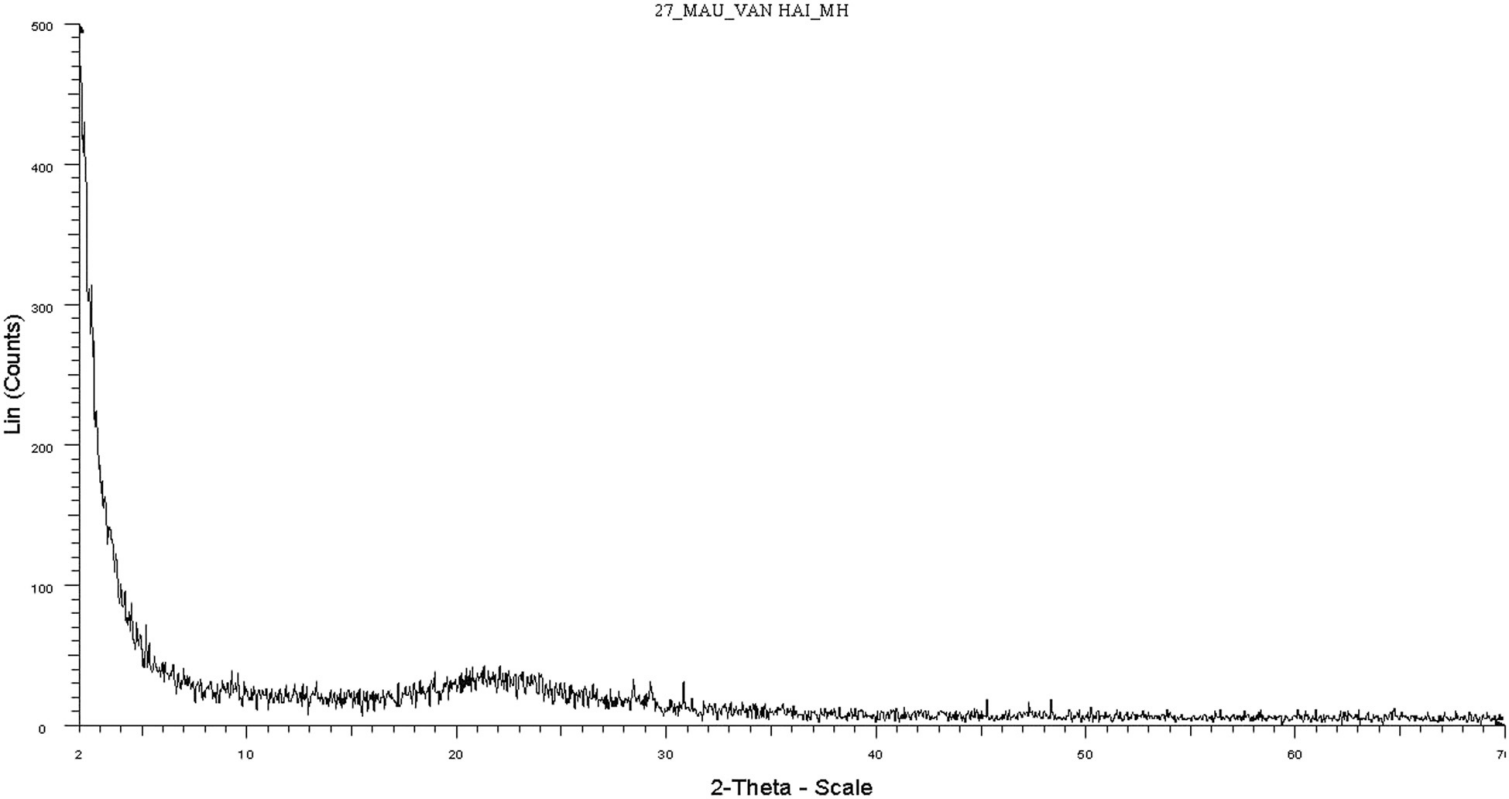
XRD diagram of silica nanoparticle.

In addition, the characteristic peak corresponding to the silica crystalline structure was not clearly observed at 2*θ* = 22° in the XRD diagrams of Figure 9, indicating that the samples are nearly amorphous.

## Conclusions

RHA material was successfully synthesized from the abundant Vietnamese rice husk. A new synthetic method for spherical silica nanoparticles using RHA as the silica source and CTAB as the surfactant via the sol–gel technique in water/butanol was investigated. This method is a simple and effective route for preparing ultrafine powders on a nanometer scale and with a homogeneous particle size distribution. The specific surface area is reached at 340 m^2^/g, and the silica product obtained is amorphous. This leads to the low-cost production of silica nanoparticles for various practical applications such as pollution treatment, nanocomposite materials, etc. Furthermore, using this source for the production of RHA provides a way to solve the waste problem of rice husk pollution in the Mekong Delta of Vietnam.

## Competing interests

The authors declare that they have no competing interests.

## Authors’ contributions

VHL, CNHT, and HHT have worked equally in all results presented in this paper. All authors read and approved the final manuscript.

## Authors’ information

VHL graduated and received his Bachelor of Science in Organical Chemistry in 2005, and after that, he received his M.S. in Physical Chemistry in 2011 from the University of Science, HoChiMinh City, Vietnam. His research interests include nanomaterials and polymers.

CNHT is currently the Vice Dean of the Faculty of Materials Science, University of Science-National University of HoChiMinh City, Vietnam. He graduated with the degree B.S. in Physical Chemistry from the University of Science, HoChiMinh City, Vietnam, in 2004. He received his M.S. in Physico-chemistry of Materials from the University of Maine, Le Mans, France, in 2005 and received his Ph.D. in Materials Science and Engineering from the University of Savoie, Chambéry, France, in 2008. His research interests include polymers, nanocomposites based on polymers, and biodegradable polymers.

HHT is an associate professor in the Faculty of Chemistry, University of Science, Vietnam National University in HoChiMinh City, Vietnam. He is also the Chief of the Polymer Laboratory, Faculty of Chemistry, University of Science, Vietnam National University in HoChiMinh City, Vietnam. Moreover, he is visiting Professorships at Université du Maine, Le Mans, France; Université de Savoie, France; Polytechnic University - Vietnam National University in HoChiMinh City, Vietnam; and Can Tho University, Vietnam. He received his Bachelor of Chemistry degree from the Faculty of Sciences, Saigon University, Vietnam, in 1971 and then received his Master of Science in Organic Physical Chemistry from the Faculty of Sciences, Saigon University in 1972. He graduated with a Ph.D. degree from the University of HCM City in 1992. He was the Dean of the Faculty of Chemistry, University of Science-Vietnam National University in HoChiMinh City, Vietnam, from 2002 to 2007. His research interests include modification of natural polymers (rubber, chitosan, etc.), natural or synthetic polymer-controlled degradation, synthesis of systems containing free and/or linked plant growth stimulator molecules in rubber/polymer matrix, living polymers, polymer blends, composite materials, nanocomposites, and graphene.
